# Early Life Circumstances and Cognitive Aging: Longitudinal Evidence From China Health and Retirement Study

**DOI:** 10.1093/geroni/igaa057.202

**Published:** 2020-12-16

**Authors:** Zhuoer Lin, Xi Chen

**Affiliations:** Yale University, New Haven, Connecticut, United States

## Abstract

Objectives: This study examines the long-term relationship between early life circumstances and later life cognitive aging. In particular, we differentiate the long-term effects of early life circumstances on level of cognitive deficit and rate of cognitive decline. Methods: Cognitive trajectories were measured using three waves of China Health and Retirement Longitudinal Surveys (CHARLS 2011-2015). Linear mixed-effect model was used to decompose the individual level of cognitive deficit and rate of cognitive change in a sample of Chinese middle-aged and older adults 45-90 years of age (N=6,700). These two dimensions of cognition were matched to four domains of early life circumstances using CHARLS Life History Survey (2014), including childhood socioeconomic status, neighborhood environment, social relationships and health conditions. Their associations were examined by linear regressions. Stratification analysis was further conducted to investigate the mediating effect of education on early life circumstances and cognitive aging. Results: Childhood socioeconomic status, childhood friendship and early life health conditions were significantly associated with both the level of cognitive deficit and rate of decline. In contrast, the community environment, including childhood neighborhood safety and social cohesion, only affected the baseline level of cognitive deficit; and childhood relationship with parents only affected the rate of cognitive decline. Moreover, education was found to be a mediating factor of these relationships. Conclusion: Exposure to disadvantaged early life circumstances have significant negative effects on later life cognitive deficit as well as rate of cognitive decline. Nevertheless, these long-term impacts can be partially ameliorated by higher educational attainment.

